# Bilirubin as a Predictor of Complicated Appendicitis in a District General Hospital: A Retrospective Analysis

**DOI:** 10.7759/cureus.29036

**Published:** 2022-09-11

**Authors:** Sattam A Halaseh, Marcos Kostalas, Charles Kopec, Abdullah Nimer

**Affiliations:** 1 General and Colorectal Surgery, Torbay Hospital, Torbay and South Devon NHS Foundation Trust, Torquay, GBR; 2 Upper Gastrointestinal Surgery, Torbay and South Devon NHS Foundation Trust, Torquay, GBR; 3 General and Colorectal Surgery, Torbay and South Devon NHS Foundation Trust, Torquay, GBR; 4 Faculty of Medicine, University of Jordan, Amman, JOR

**Keywords:** surgical acute abdomen, systemic inflammation marker, complicated appendicitis, diagnosis of acute appendicitis, bilirubin

## Abstract

Aims

The objectives of the study were to establish the function of bilirubin as a novel diagnostic tool for predicting complex appendicitis and to compare the impact of other variables such as white blood cell count (WCC), C-reactive protein (CRP), and neutrophil.

Methods

This retrospective, single-center cohort analysis included all patients admitted to Torbay General Hospital in Torquay, United Kingdom, between January 2020 and December 2020 with a clinical diagnosis of acute appendicitis. In addition to serum CRP, WCC, and neutrophil, serum bilirubin and other liver enzymes were obtained from the patients’ blood on admission.

Results

The quantitative analysis included 174 patients from the group that remained. The large majority of the sample consisted of adults and males (77% and 51.7%, respectively). Overall, 49.4% of participants in the study were diagnosed with complicated appendicitis; 74.7% of adults had complicated appendicitis, with 58.6% being male. In 68.6% of cases, perforated appendicitis was the most prevalent form of complicated appendicitis. Patients with complicated appendicitis had significantly higher WCC, neutrophil, and CRP levels than those with uncomplicated appendicitis (14.15 vs. 12.88, p = 0.016; 11.63 vs. 10.19, p = 0.007; and (89.28 vs. 40.65, p = 0.0001, respectively).. The significantly greater alkaline phosphatase and total serum bilirubin discrepancies were observed in patients with complicated appendicitis. There were statistically significant differences in the means of the patients: (18.46 vs. 10.98, p = 0.0001 and 110.64 vs. 102.24, p = 0.033).

Conclusion

Serum bilirubin is a crucial diagnostic aid for determining the existence of complicated appendicitis.

## Introduction

Acute appendicitis is one of the leading causes of acute abdomen. Around 7% of the population may contract this condition at some point in their lives, and up to 20% of those affected will suffer a perforation as a result [1]. In the United Kingdom, almost 50,000 appendectomy procedures are carried out annually [2]. Although anyone is susceptible to acute appendicitis, it often strikes between 10 and 20 years of age, with males being disproportionately affected by this condition [3].

In most cases, symptoms and physical examination results are all that are needed to make a diagnosis of acute appendicitis. Many other scoring systems have been developed to aid in the diagnosis; the most well-known of them is called the Alvarado score, which takes into account both clinical symptoms and laboratory findings. Diagnostic scores for appendicitis include special emphasis on patient history, physical examination findings, and laboratory results (such as white blood cell count [WCC] and/or C-reactive protein [CRP]) [4,5]. Recently, imaging techniques such as ultrasound and computed tomography (CT) have been incorporated into the diagnostic process, increasing sensitivity but also bringing their own set of limitations with them. Contrasted with the widespread use of CT, which would increase costs and lead to excessive radiation exposure, ultrasonic scan results require an experienced diagnostician for accurate, thorough interpretation [6,7].

Because of this, a simple, affordable, and readily available test that is diagnostic for acute appendicitis and its consequences, in addition to clinical symptoms, is required for making the diagnosis, anticipating its extent and severity, and deciding on appropriate care. Previous research has already established the importance of certain blood markers such as CRP and WCC [8,9]. However, there is no correlation between a higher WCC and the likelihood of developing complications from uncomplicated versus complicated appendicitis [10].

Recent studies have looked at the correlation between an increase in blood total bilirubin concentration and the progression to complicated acute appendicitis in an effort to identify a diagnostic marker for this condition [11,12]. Serum bilirubin elevations can be accounted for by the spread of Gram-negative bacteria from the appendix into the portal system and liver, where they cause endotoxin-mediated disruptions of bile duct bilirubin excretion [13,14]. In addition, further research has found that septic-induced hemolysis is a major factor contributing to the elevation of serum bilirubin in acute complicated appendicitis [15].

This study aimed to evaluate total blood bilirubin levels as a preoperative diagnostic measure of acute complicated appendicitis in a cohort of patients with diagnosed acute appendicitis who subsequently underwent appendectomy.

## Materials and methods

Project setting

This retrospective, single-center cohort analysis comprised 234 patients with a clinical diagnosis of acute appendicitis who were admitted by the Emergency Department or the Acute Surgical Receiving Unit to Torbay General Hospital in Torquay, United Kingdom, between January 2020 and December 2020.

Patient selection

**Figure 1:**
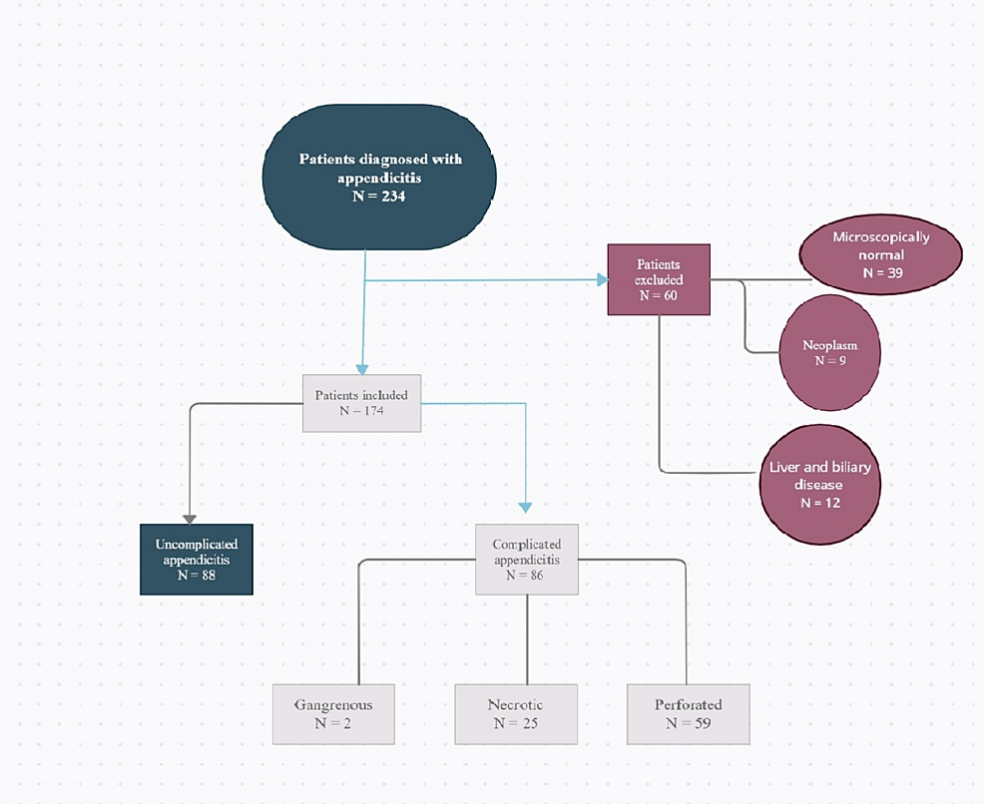
A flow chart showing patient selection.

Patients who had histological, CT scan, or ultrasound scan evidence of acute appendicitis met the inclusion criteria. Individuals with clinical suspicion of acute appendicitis but no findings on histopathological examination and patients with evidence of neoplastic abnormalities on histopathology were excluded from the study. Also, patients with known liver diseases such as cirrhosis, Gilbert’s disease, or other documented biliary/hemolytic disease were excluded. The initial case review resulted in the exclusion of 60 patients: 39 with a microscopically normal appendix, 12 with a history of liver and biliary disease, and 9 with a diagnosis of malignancy based on appendix histopathological examination. Refer to Figure 1 for a flow chart of patient selection.

Data collection and laboratory methods

Histopathological examination of all appendectomy specimens was conducted at the pathology lab. At the time of admission, venous blood samples were taken for analysis of CRP, white blood cell count (WBC), total serum bilirubin (TB), alanine transaminase (ALT) levels, aspartate aminotransferase (AST) levels, and alkaline phosphatase (ALP) levels. Our institution's laboratory reported that WBC levels below 10,000/mm^3^, TB levels below 20 mmol/L, and CRP levels below 5 mg/mL were all within normal ranges. An individual's age and gender were among the clinical and demographic details we submitted. The study's participants were divided into two groups: those with uncomplicated appendicitis and those with complicated appendicitis, as determined by the final pathology reports, and radiological, and surgical findings.

Definition

The established definition of uncomplicated appendicitis was the existence of histopathological or radiological proof of simple, localized, or purulent appendicitis. Patients with periappendiceal abscess development, peritonitis, gangrenous appendicitis, or radiologically proven gangrenous appendicitis are all considered to be part of the complicated group.

Statistical analysis

The patient information was recorded in Microsoft Office Excel 2019 and subsequently integrated into SPSS (IBM Corp., Armonk, NY). IBM SPSS Statistics for Windows, Version 26.0 (IBM Corp.) was utilized for data analysis. For descriptive statistics, simple counts and percentages were employed, coupled with the chi-square test to demonstrate differences in means. Independent samples Mann-Whitney U test was employed to predict lab values of complicated appendicitis. Furthermore, the receiver operating characteristic (ROC) curve and area under the curve (AUC) were utilized to predict the sensitivity and specificity of laboratory results reported to have a strong relation with complicated appendicitis. For all tests, a 95% level of confidence and a p-value of less than 0.05 were considered significant.

## Results

At our institution, a total of 234 individuals were given a clinical diagnosis of appendicitis between January 1, 2020, and December 31, 2020.

The quantitative analysis comprised 174 patients from the remaining group. Adults and males made up the vast majority (77% and 51.7%, respectively) of the sample. The average age of the cohort was 37.2 years. Laparoscopic surgery accounted for 86.2% of all procedures, with patients staying an average of 3.08 days in the hospital as a result. Patients' demographics are listed in Tables 1, 2.

**Table 1:** Patient demographics including gender, age groups, complicated appendicitis, and treatment approach.

Variable	Response	Frequency	Percentage
Gender	Male	90	51.7%
	Female	84	48.3%
Age group	Adults	134	77%
	Pediatrics	40	23%
Complicated appendicitis	Yes	86	49.4%
	No	88	50.6%
Treatment approach	Open appendicectomy	14	8%
	Laparoscopic appendectomy	150	86.2%
	Laparoscopic converted to open	6	3.5%
	Conservative	4	2.3%

**Table 2:** Patient demographics including age, and length of stay.

Variable	Mean
Age (year)	37.2
Length of stay (day)	3.08

In the analysis, 49.4% of patients were diagnosed with complicated appendicitis. Adults with complicated appendicitis represented 74.7%, with 58.6% being male. Perforated appendicitis was the most common type of complicated appendicitis, accounting for 68.6% of cases, followed by necrotic appendicitis, which accounted for 29.1% of complicated appendicitis cases, and finally, gangrenous appendicitis, which accounted for 2.3% of complicated appendicitis cases. Table 3 shows information about the incidence of complicated appendicitis among the cohort group.

**Table 3:** The incidence of complicated appendicitis among the cohort group.

Complicated appendicitis classification		Perforated	Necrotic	Gangrenous
Total	87	59 (68.6%)	25 (29.1%)	2 (2.3%)
Gender	Male	33	17	1
	Female	27	8	1
Age group	Adults	47	17	1
	Pediatrics	12	8	1
Average length of stay (day)		3.78	4.89	5

We proceeded by comparing the pro-inflammatory markers across individuals diagnosed with uncomplicated and complicated forms of appendicitis. Each of these patients was tested for CRP, neutrophils, and WCC levels. We compared the mean WCC, neutrophil, and CRP counts between the two groups. Patients with complicated appendicitis exhibited substantially higher WCC, neutrophil, and CRP levels compared to those with uncomplicated appendicitis (14.15 vs. 12.88, p =0.016, 11.63 vs. 10.19, p = 0.007, and 89.28 vs. 40.65, p = 0.0001, respectively). See Table 4 for the results.

**Table 4:** Independent samples Mann-Whitney U Test showing the difference in lab value means between complicated and uncomplicated appendicitis cases. CRP, C-reactive protein; WCC, white blood cell count

	Complicated mean value	Uncomplicated mean value	p-Value
CRP (mg/L)	89.28	40.65	0.000
WCC (x10^9^/L)	14.32	12.88	0.016
Neutrophil (x10^9^/L	11.43	10.19	0.007

We analyzed the correlation between bilirubin and other liver function tests and the development of complicated appendicitis. The levels of TB, AST, ALT, and ALP in each of these individuals were evaluated. We analyzed the mean values of those tests between the uncomplicated and complicated patient groups using the Mann-Whitney U test. ALP and TB differences were significantly greater in patients with complicated appendicitis. There was a statistically significant difference in the patients' means (18.46 vs. 10.98, p = 0.0001 and 110.64 vs. 102.24, p = 0.033). Although the mean AST and ALT were greater in the complex group, there was no statistically significant difference between the two groups. See Table 5 for the results.

**Table 5:** Independent samples Mann-Whitney U test showing the difference in lab value means between complicated and uncomplicated appendicitis case. AST, aspartate aminotransferase; ALT, alanine transaminase; ALP, alkaline phosphatase; TB, total serum bilirubin

	Complicated mean value	Uncomplicated mean value	p-Value
AST (IU/L)	27.46	22.24	0.089
ALT (IU/L)	26.77	21.74	0.212
ALP (IU/L)	110.64	102.24	0.033
TB (umol/L)	18.46	10.98	0.000

**Figure 2:**
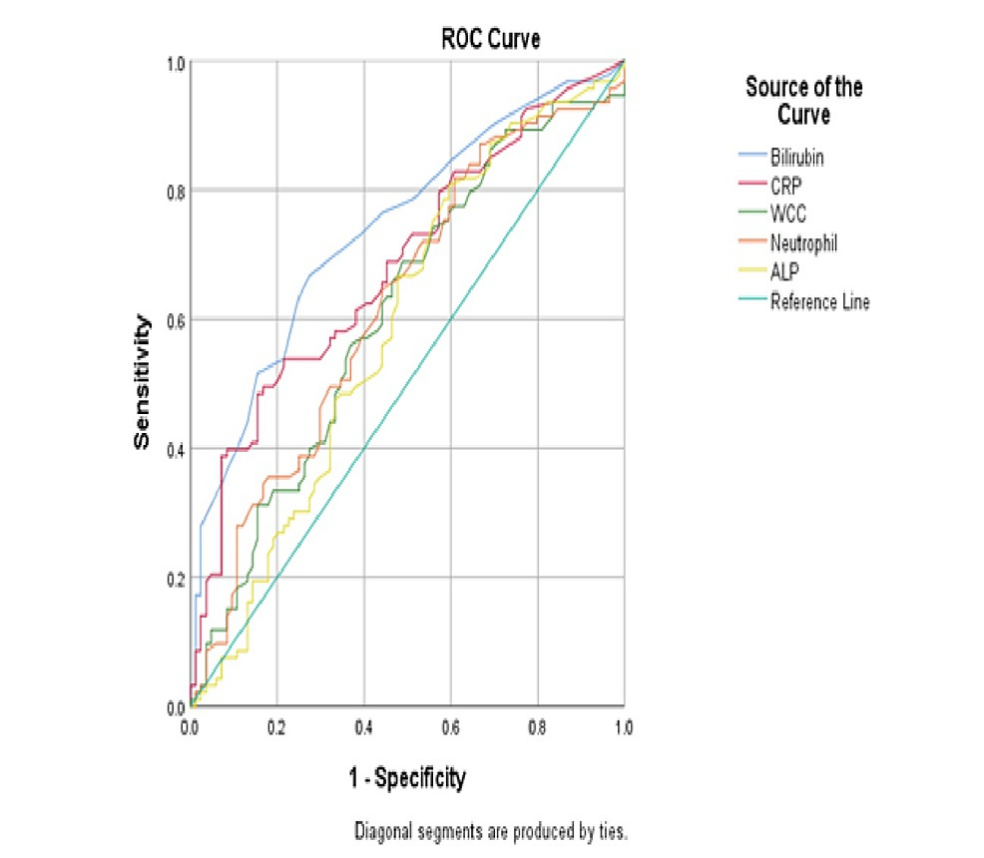
ROC curves to distinguish between uncomplicated and complicated cases of acute appendicitis. ROC, receiver operating characteristics

The WCC, neutrophil, CRP, TB, and ALP were tested for sensitivity, specificity, and accuracy. The threshold value of the aforementioned components was determined using ROC curves to distinguish between uncomplicated and complicated cases of acute appendicitis (Figure 2). In comparison to the other tests, the TB had the highest accuracy at 73.9% (P value <0.001). The sensitivity of TB was 73.3%, which was the highest of all of the tests. In addition, CRP with a proposed cutoff value of 38.5 had the best specificity compared to TB at 62% vs. 61%. Table 6 shows the results.

**Table 6:** The sensitivity, specificity, and accuracy of CRP, WCC, neutrophils, ALP, and total serum bilirubin. AUC, area under the curve; CRP, C-reactive protein; WCC, white blood cell count; ALP, alkaline phosphatase

Variable	AUC	p-Value	95% CI lower bound	95% CI upper bound	Suggested cut-off value	Sensitivity	Specificity
CRP	0.68	<0.001	0.61	0.76	38.5	61.1%	62%
WCC	0.620	0.015	0.507	0.693	12.7	68.2%	54%
Neutrophils	0.612	0.004	0.530	0.715	10.1	67%	53%
ALP	0.589	0.041	0.504	0.674	78	67%	53%
Total serum bilirubin	0.739	<0.001	0.666	0.811	11.5	73.3%	61%

## Discussion

As far as appendicitis is concerned, bilirubin is not a generally recognized marker. Raised bilirubin levels have been shown in prior studies to be a good and specific marker for perforated appendicitis [16,17]. Among the adult surgical population, elevated bilirubin levels are frequently the consequence of liver or gallbladder disorders [18]. Hepatic dysfunction caused by sepsis has been extensively studied [19]. In acute appendicitis, the rise of serum bilirubin is thought to be caused by microorganisms and bacterial toxins migrating via the portal vein and eventually being metabolized by the liver [20]. The dysfunctionality of the hepatocytes, driven by the toxic nature of the bacterial products and the products of the inflammatory response, influences bilirubin excretion [21]. When the appendix wall is damaged, microorganisms and endotoxins can migrate out of the appendix lumen and into the portal system. After that, the inflammatory cytokines potentially end up in the liver, where they could trigger intrahepatic cholestasis. Our findings of rising bilirubin levels with increasing appendicitis severity are consistent with previous studies showing that Escherichia coli endotoxin produces dose-dependent cholestasis [22].

Hyperbilirubinemia and vermiform appendix inflammation have been studied extensively in recent years, with some research identifying bilirubin as a unique marker for appendiceal perforation. Elevated bilirubin levels have been linked to complicated appendicitis, as reported by many studies ([12,16,23,24]). Among those trials, TB sensitivity ranged from 49 to 70%, with specificity hovering between 70% and 86%. The pooled sensitivity and specificity of this study were 73.3%, and 61%, respectively.

It is still the clinical presentation of the patient and the discretion of the emergency surgeon that determine whether or not the patient has appendicitis. Blood tests (including WBC and CRP) and imaging are the most typical ancillary diagnostic techniques. Although imaging tests such as CT scans improve diagnosis accuracy, they are underutilized because of safety concerns about ionizing radiation for young patients who tend to suffer from appendicitis [25]. Affordability might be an issue as well. This study confirms that increased bilirubin levels can indicate complicated appendicitis with better accuracy and sensitivity than WCC and CRP (73.9 vs. 62% vs. 68%, and 73.3 vs. 68.2% vs. 61.1%, respectively).

Limitations

This study has methodological shortcomings. First, it is a retrospective analysis with a reasonable number of participants. Second, the study's retrospective design prevented researchers from dissecting total bilirubin concentration (unconjugated and conjugated). The potential inclusion of people with Gilbert's syndrome is another area of concern. Finally, the patient's delayed presentation of symptoms may have affected the blood test findings.

## Conclusions

Surgeons often have difficulty making an accurate diagnosis of acute appendicitis; nevertheless, our results suggest that a serum bilirubin level is a strong indicator of the presence of complicated appendicitis. TB, in combination with others such as WCC, CRP, and clinical presentation, is more sensitive and specific in identifying patients who would develop complicated appendicitis. Future research using a larger sample is necessary to draw firmer conclusions on this question.
